# Draft Genome Sequence of a Bovine Enterovirus Isolate Recovered from Sewage in Nigeria

**DOI:** 10.1128/MRA.01466-18

**Published:** 2018-12-13

**Authors:** T. O. C. Faleye, O. M. Adewumi, O. A. Olayinka, E. Donbraye, B. Oluremi, U. E. George, O. A. Arowolo, E. C. Omoruyi, M. I. Ifeorah, A. O. Oyero, J. A. Adeniji

**Affiliations:** a Department of Virology, College of Medicine, Faculty of Basic Medical Sciences, University of Ibadan, Ibadan, Nigeria; b Department of Microbiology, Faculty of Science, Ekiti State University, Ado-Ekiti, Nigeria; c Department of Medical Microbiology and Parasitology, Obafemi Awolowo University, Ile-Ife, Nigeria; d Department of Pharmaceutical Microbiology, Faculty of Pharmacy, University of Ibadan, Ibadan, Nigeria; e Viral Vaccines Production Division, National Veterinary Research Institute, Vom, Plateau State, Nigeria; f Institute of Child Health, College of Medicine, University of Ibadan, Ibadan, Nigeria; g Department of Medical Laboratory Sciences, Faculty of Health Sciences and Technology, University of Nigeria, Nsukka, Nigeria; h WHO National Polio Laboratory, University of Ibadan, Ibadan, Nigeria; Indiana University, Bloomington

## Abstract

We describe the draft genome of a bovine enterovirus (EV) isolate recovered from sewage in Nigeria. This isolate replicates on both RD and L20B cell lines but is negative for all EV screens in use by the Global Poliovirus Eradication Initiative (GPEI). It contains 7,368 nucleotides (nt) with 50.2% G+C content and an open reading frame (ORF) with 6,525 nt (2,174 amino acids).

## Abstract (precis)

We describe the draft genome of a bovine enterovirus (EV) isolate recovered from sewage in Nigeria. This isolate replicates on both RD and L20B cell lines but is negative for all EV screens in use by the Global Poliovirus Eradication Initiative (GPEI).

## ANNOUNCEMENT

Enteroviruses are members of the genus Enterovirus (EV), family *Picornaviridae*, order *Picornavirales*. Poliovirus (PV) is the type member of the genus and, courtesy of the Global Poliovirus Eradication Initiative (GPEI), is isolated in about 150 specialized laboratories globally. The laboratories use RD (of human origin) and L20B (engineered mouse cells expressing the poliovirus receptor) cell lines for PV isolation (1, 2). All isolates that grow on both cell lines are assumed to be polioviruses and subsequently subjected to molecular identification (3). Here, we describe the genome of an isolate, EV_NGR_2017, that grew on both cell lines but is not poliovirus.

The isolate was recovered from a sewage-contaminated water sample collected in Borno State, Nigeria, in 2017. It was inoculated into (and replicated on) both RD and L20B cell lines but was not poliovirus. The genome of the isolate was extracted using a total RNA extraction kit (Jena Bioscience, Germany) and used for cDNA synthesis as previously described (4). The single-stranded cDNA was shipped to a commercial facility (MR DNA, TX, USA), where library preparation and genome sequencing and assembly were done. Library preparation was done using the TruSeq RNA LT sample preparation kit (Illumina) following the manufacturer’s recommendations. Sequencing was done paired end for 300 cycles using the MiSeq system (Illumina), assembly of the 7.8 million reads was done using Newbler (Roche), and annotation of the Enterovirus *E* (EV-E) genome was done by aligning it (using MEGA5 software [5]) with previously characterized and annotated EV-E genomes in GenBank.

Precisely 7,810,328 reads were generated. The draft genome is 7,368 nucleotides (nt) long, with a G+C content of 50.2%, and was assembled from 6,862 (0.09%) reads. The 5′ untranslated region (5′-UTR), open reading frame (ORF), and 3′-UTR contain 800 nt, 6,525 nt (2,174 amino acids [aa]), and 43 nt, respectively. The ORF encodes 1 polyprotein that can be cleaved into 3 (P1 [2,517 nt, 839 aa], P2 [1,737 nt, 579 aa], and P3 [2,271 nt, 756 aa]) and subsequently into the 11 proteins encoded in EV genomes. A BLASTn search of the draft genome against the GenBank database showed it to be most closely related to strain BEV IS1/Bos taurus/JPN/1990 (GenBank accession number LC150009) (an enterovirus recovered from a cow in Japan in 1990). A BLASTn search of the VP1 gene, however, showed it to be most closely related to isolate 56/59/1 (DQ092778) (an enterovirus recovered from a cow in Germany in 1999). In the VP1 gene, EV_NGR_2017 and BEV IS1/Bos taurus/JPN/1990 are 71.6% and 93.7% similar in nucleotides and amino acids, respectively. On the other hand, EV_NGR_2017 and 56/59/1 (DQ092778) are 71.7% and 91.4% similar in nucleotides and amino acids, respectively.

Classically, bovine enteroviruses are cultured in Mardin-Darby bovine kidney (MDBK) cells (6, 7). Therefore, investigations of the biological basis of EV_NGR_2017 replication in both human (RD) and mouse (L20B) cell lines and the implications for zoonosis are needed. Kaundal et al. (8) recently described unidentifiable isolates from sewage in India that replicated in both RD and L20B cell lines but were not polioviruses. These isolates might also be bovine enteroviruses.

### Data availability.

The draft genome assembly and raw reads have been deposited in GenBank and SRA under the accession numbers MH719217 and PRJNA493004, respectively.
